# President's Address

**Published:** 1906-08

**Authors:** 


### PRESIDENT'S ADDRESS.

(Florida State Dental Society, 1906.)

In pursuance of the time-honored custom, the duty of making a few introductory remarks falls upon the presiding officer, and in presenting this my address I have the pleasure, as well as the honor of welcoming you to this our twenty-third annual meeting, and I trust it will be a session both pleasant and profitable to us all.

At the last meeting when the honor of presiding for this year was conferred upon me I tried as best I could to express my appreciation, but for fear I failed at that time to express my true feelings, I wish to again extend to you my thanks for the great honor, and assure you that in the future I shall look with much pleasure back to the time when I was permitted to act as your president.

One of the duties incumbent upon the position to which you have chosen me is to preside at the sessions and carry out the program as prescribed by our by-laws and arranged by our various committees. I feel that each member will join me in an unstinted expression of thanks to the committeemen and officers who have exerted their efforts to arrange for our consideration a series of interesting features, which I am sure will be of benefit to each and every one of us here present. This can be derived by individual interest, and the various ideas being thoroughly set forth and discussed. In presiding I shall endeavor to encourage friendly discussion as long as there is any interest manifested.

We have up to the present time 118 active and 8 honorary members, making a total of 126. A number of these are in arrears and are subject to being stricken from the membership roll. We have in the state between 200 and 225 dentists now practicing, so you will see by these figures that we have little more than half of them in the society. We should have more! I think it the duty of each and every member to use their efforts to secure the application of the men practicing in their respective localities, who are not identified with the society, and present them to the executive committee for investigation. I believe by doing so we can create to a great extent more of the brotherly feeling which should exist within the hearts of those who are most luke-worm now, and perhaps eliminate many of the petty jealousies which always exist in competition.

This society has followed in the lines set by the founders and accomplished much for the betterment of both practitioner and patient. It has raised the standard of the profession to a great extent, but there is still many good things in store for us. Let us endeavor to put forward our best efforts, preparing for and carrying out the session, and thus make more rapid strides upward and onward. We should make our meetings as near a post-graduate school as possible, by having good practical clinics, interesting papers, and advancing new theories, thus creating more enthusiasm and discussion. This appears to me to be the simplest way to make our meetings something to be looked forward to with much pleasure.

I think we should have more practical clinical work at our meetings. Some effort has been exerted by a few of our members to have something good along this line at this meeting. The program announces several interesting demonstrations. I hope they will be the means of having more in the future. We are greatly indebted to those who have contributed clinics to us in the past, as no doubt much of the skill of many of our members has been derived from clinics in the past. We can teach, or be taught, clinically much easier than by any other method. We can read a description of performing an operation, but it will be impossible to grasp all the details in every case. The way it is set forth on paper is sometimes confusing, but if we can have an ocular demonstration of it, we take it at once No two men have exactly the same method of performing an operation, although the results may be practically the same, and it is the little points of detail in manipulation, which we get from each other, which are of greatest help to us. They assist us over the rough places and it also broadens our minds and prevents us from getting into ruts and grooves. I would recommend that we discuss and devise some way whereby we can create a fund to be used for clinics.

I also think we have not devoted enough time to paper. We should endeavor to encourage the spirit of writing papers, and especialy when we are appointed on a Committee, the members should either prepare or secure something on their branches. They, should at least make some effort to bring before the society "something new" on their respective branches, and not come in when the committee is called with this "no report". I hope we will have good reports from each and every committee at this session and at future sessions.

There has been a few instances of disregard for our code of ethics, which have come to my notice within the past yea.. I feel that enough has been said along this line by my predecessors in office, still I cannot refrain from a word or two in condem..atin of such. The society is what its members make it, their professinal conduct reflects upon it either credit or discredit, and no Dentist should by an unprofessional act tarnish the fair name of the society to which he belongs.

The standard of Dentistry as a profession, has been raised until to-day we can hold up our heads with pride in our calling and gentlemen we should do nothing, as individuals,or as a society, to lower it.

Value yourself and your profession and others will value you, and if you prize your profession as you should, you will be free from jealousy and despise the ways, which seem sometimes to promise to raise you in the estimation of the community at the expense of your brother practitioner.

In conclusion I extend to the visiting brethren a cordial welcome along the privileges of the floor during our session, and ask them to join with us in our discussions.

I thank you for your attention.

